# The Influence of pCO_2_ and Temperature on Gene Expression of Carbon and Nitrogen Pathways in *Trichodesmium* IMS101

**DOI:** 10.1371/journal.pone.0015104

**Published:** 2010-12-06

**Authors:** Orly Levitan, Stefanie Sudhaus, Julie LaRoche, Ilana Berman-Frank

**Affiliations:** 1 The Mina and Everard Goodman Faculty of Life Sciences, Bar Ilan University, Ramat Gan, Israel; 2 Leibniz Institute of Marine Sciences at Kiel University (IFM-GEOMAR), Kiel, Germany; University College Dublin, Ireland

## Abstract

Growth, protein amount, and activity levels of metabolic pathways in *Trichodesmium* are influenced by environmental changes such as elevated pCO_2_ and temperature. This study examines changes in the expression of essential metabolic genes in *Trichodesmium* grown under a matrix of pCO_2_ (400 and 900 µatm) and temperature (25 and 31°C). Using RT-qPCR, we studied 21 genes related to four metabolic functional groups: CO_2_ concentrating mechanism (*bicA1, bicA2, ccmM, ccmK2, ccmK3, ndhF4, ndhD4, ndhL, chpX*), energy metabolism (*atpB*, *sod*, *prx*, *glcD*), nitrogen metabolism (*glnA*, *hetR, nifH*), and inorganic carbon fixation and photosynthesis (*rbcL, rca, psaB, psaC, psbA*). *nifH* and most photosynthetic genes exhibited relatively high abundance and their expression was influenced by both environmental parameters. A two to three orders of magnitude increase was observed for *glnA* and *hetR* only when both pCO_2_ and temperature were elevated. CO_2_ concentrating mechanism genes were not affected by pCO_2_ and temperature and their expression levels were markedly lower than that of the nitrogen metabolism and photosynthetic genes. Many of the CO_2_ concentrating mechanism genes were co-expressed throughout the day. Our results demonstrate that in *Trichodesmium*, CO_2_ concentrating mechanism genes are constitutively expressed. Co-expression of genes from different functional groups were frequently observed during the first half of the photoperiod when oxygenic photosynthesis and N_2_ fixation take place, pointing at the tight and complex regulation of gene expression in *Trichodesmium*. Here we provide new data linking environmental changes of pCO_2_ and temperature to gene expression in *Trichodesmium*. Although gene expression indicates an active metabolic pathway, there is often an uncoupling between transcription and enzyme activity, such that transcript level cannot usually be directly extrapolated to metabolic activity.

## Introduction

The marine filamentous N_2_ fixing (diazotroph) cyanobacteria *Trichodesmium* spp. form extensive blooms contributing 25 to 50% of the estimated rates of N_2_ fixation in the oligotrophic subtropical and tropical oceans [1]. *Trichodesmium's* dominant role in carbon and nitrogen cycling has prompted investigations examining the effects of rising sea surface temperatures and elevated atmospheric pCO_2_ (leading to ocean acidification) on the growth and abundance of this organism.

Elevated pCO_2_ supports enhanced N_2_ fixation and growth rates in *Trichodesmium* [2]–[7]. These trends are further accentuated when elevated pCO_2_ and higher temperatures are combined [3], [5]. The higher N_2_ fixation and growth rates are enabled via flexible phosphorus stoichiometry, changes in the activity of the CO_2_ concentrating mechanism (CCM), and modified protein activity [4]–[8].

In *Trichodesmium*, as in other cyanobacteria, metabolic pathways (e.g. respiration, photosynthesis, Ci fixation, N_2_ fixation, and combined nitrogen assimilation) share cellular complexes such as plastoquinone (PQ) pool, succinate dehydrogenase and ferredoxin [9]–[11]. *Trichodesmium's* unique metabolism allows oxygenic photosynthesis and oxygen-sensitive N_2_ fixation to occur concurrently during the photoperiod via a complex spatial-temporal separation of these processes [11]–[15]. Photosynthetic activity in *Trichodesmium* is coupled with CCM activity. PSII driven electron transport is responsible for generating energy needed to pump HCO_3_^−^ into the cell. This HCO_3_^−^ is subsequently converted to CO_2_ to be used by the ribulose-1,5-bisphosphate carboxylase oxygenase (RubisCO) within the carboxysomes [6]. Regulation of the photosynthetic and N_2_ fixation processes occurs at the transcription, translation, and post-translational (activity) levels [5], [8], [10], [12], [13], [16], [17]. While elevated pCO_2_ and temperature resulted in higher growth rates, higher N_2_ fixation rates, and higher C∶P ratios, photosynthesis, protein pools, and total cellular allocation of carbon and nitrogen were not significantly affected [5]. Importantly, the abundance of nitrogenase and glutamine synthetase (mediating combined nitrogen assimilation) did not increase in parallel to the increased N_2_ fixation rates, implying that environmental factors can allow higher reaction turnover rates through the same protein amounts [5], [8]. In addition, our previous study showed that pCO_2_ changed the mRNA diel expression patterns, but not the abundance, of five genes (*nifH*, *glnA*, *hetR*, *psbA*, and *psaB*), resulting in a more synchronized expression pattern under elevated pCO_2_ [5]. We therefore decided to check the combined effect of pCO_2_ and temperature on the expression levels of 21 genes of interest (GOI) representing key metabolic aspects in *Trichodesmium*, as part of *Trichodesmium'*s acclimation response.

Genomic analyses demonstrate that *Trichodesmium* (IMS101) has a partial suite of CCM components ([18], [19]; http://genome.jgi-psf.org/finished_microbes/trier/trier.home.html). Accordingly, *Trichodesmium* possesses β-carboxysomes, a cellular compartment containing RubisCO, and a low-affinity, high-flux HCO_3_^−^ uptake system called BicA [18]–[20]. *Trichodesmium* also has a specialized NADPH dehydrogenase, NDH-I_4_, which acts as a low-affinity CO_2_ uptake system, converting CO_2_ to HCO_3_^−^ using the ChpX protein [18]. The presence of a true internal carbonic anhydrase (CA) was not found in the genome and direct measurements by means of ^18^O_2_ exchange method [21] revealed only a low activity, close to the detection limit of the method [6]. Yet, there is a distinct possibility that the N-terminal domain of the essential β-carboxysomal *ccmM* gene found in *Trichodesmium* can act as a γ–CA in an oxidized β-carboxysome interior as was observed in *Thermosynechococcus elongatus* [19], [22], [23].

Currently, there is no genetic system for *Trichodesmium* transformations, limiting the physiological study of CCM activity to the examinations of fluxes of inorganic carbon (Ci) and O_2_ [6]. Here we present the expression and abundance of genes related to CCM (*bicA1, bicA2, ccmM, ccmK2, ccmK3, ndhF4, ndhD4, ndhL* and *chpX*), energy metabolism (*atpB, sod, prx, glcD*), nitrogen metabolism (*glnA, hetR, nifH*), and photosynthesis and Ci fixation (*rbcL, rca, psaB, psaC, psbA*) in *Trichodesmium* acclimated to a matrix of pCO_2_ (400 and 900 µatm) and temperature (25 and 31°C). Since diurnal regulation is essential for metabolic functions in *Trichodesmium*, we performed our measurements over the day and sampled 1, 5, 9 and 13 h after the onset of light. The sampling times were chosen for time periods that represent different metabolic preferences in *Trichodesmium* [13]: time of maximal photosynthesis (1 h), maximal N_2_ fixation rates (5 h), late afternoon (9 h) and 1 h after dark induction (13 h). We compare the expression levels and patterns of these genes and look at the correlation of their coordinated expression.

## Materials and Methods

### Culturing and growth

*Trichodesmium* IMS101 stock cultures were grown in YBCII medium [24] at 25°C, 12∶ 12 light/dark cycle at ∼80 µmol photons m^−2^ s^−1^ white light and 400 µatm *p*CO_2_. Diluted batch cultures were grown in sterile square 1 L Nalgene bottles as single filaments with gentle bubbling, sufficient for preventing aggregates formation without harming the integrity of the filaments. Stock cultures were unialgal and under exponential growth the bacterial biomass was negligible and was not observed under light microscopy or by DAPI staining. Experimental cultures were enriched with CO_2_ and air mixes of 400 µatm (current) *p*CO_2_ and 900 µatm (expected 2100,) and were gradually acclimated to 31°C (1°C increase per week). Cultures were acclimated for at least 1.5–2 months before sampling. Biomass was kept under 0.2 µg chl *a* ml^−1^, thereby maintaining a low enough biomass that did not additionally influence the carbonate chemistry of the experimental setup. For more information about carbonate chemistry in similar experimental setups, see Kranz et al. [6].

### Sample collection for RNA, RNA-Extraction and reverse transcription RT-qPCR

Samples of *Trichodesmium* IMS101 were collected at 4 time points during the diurnal cycle, 1, 5, 9 and 13 h after the onset of light (the last point is 1 h after dark induction). Acclimated cultures were filtered on polycarbonate filters of 1 µm pore size; 25 mm diameter filters (Osmonics). Filters were placed in sterile DNase and RNase free centrifuge tubes and put directly into liquid nitrogen until transfer to -80°C for storage.

mRNA was extracted with the RNeasy Plant Mini Kit (Qiagen Cat.74904) according to the producers instructions. Additionally a DNase treatment was accomplished with RNase-Free DNase Set (Qiagen Cat.79254) on column during the extraction as well as with TURBO DNA free (Ambion Cat.AM1907) after the extraction, following the manufacturer's specifications for rigorous DNase treatment to remove any gDNA contamination. RNA concentration was measured with a NanoDrop ND-1000 Spectrophotometer (peqLab Biotechnologie) and quality was tested with 1% agarose gels. Reverse transcription was conducted with the QuantiTect Reverse Transcription Kit (Qiagen Cat.205311) according to the kit's manual. Each cDNA reaction contained 100 ng template RNA and was stored at −20°C until further utilization. RT-qPCR was carried out with Platinum SYBR Green qPCR SuperMix-UDG with ROX (Invitrogen Cat.11744-500) on an ABI PRISM 7000 Sequence Detection System. All three biological replicates of each sample (acclimation and time point) were measured in duplicate 25 µl reactions. The reaction mixture contained 5 µl diluted cDNA (equivalent to approx. 2 ng RNA), 12.5 µl SYBRgreen, 0.5 µl per primer (10 pmol µl^−1^) and 6.5 µl PCR water. Non-Template-Controls (NTC's) and samples with the cleaned RNA as template were run to exclude contaminations with gDNA. All NTC's and all RNA samples were below the detection limit. Cycling conditions were: 50°C for 2 min, 95°C for 2 min, 40 cycles of 95°C for 15 sec, and 60°C for 30 sec, followed by a dissociation stage of 95°C for 15 sec, 60°C for 20 sec, and 95°C for 15 sec. Primers for target genes were designed using Primer Express Software v2.0 (Applied Biosystems) and are presented, by name and function, in Table 1.

**Table 1:** Description and sequences of forward and reverse primers for our target genes.

Gene	Description	Forward Primer (5′ to 3′)	Reverse Primer (5′ to 3′)
*16s*	16s rRNA	GCGCAACCCTCGTCTTTAGTT	TTGTCACCGGCAGTCTCTTCA
*rnpB*	RNaseP	TGGTAACAGGCATCCCAGATAGATA	CGGGTTCTGTTCTCTCAACTCAA
*bicA1*	Low affinity HCO_3_^−^ transporter	GTCCTGCTGCTGGCTTATATGG	AACAGTGCTGCAAACAAACCC
*bicA2*	Low affinity HCO_3_^−^ transporter	TGTCATGCTCGGCGGAAT	TCCTAGCTGAAGAACCCCAAAA
*ccmM*	β-Carboxysome shell/gamma CA	TCGGCTTTCGTTCTACAGTTTTTAA	AACTATACATCCTTCACCAATGCG
*ccmK2*	β-Carboxysome shell	CCGAGGAGATGTTTCGGAAGTAC	CCACCATCAACTCTTTTAGCTGACTC
*ccmK3*	β-Carboxysome shell	TGCCGGAATTGCAGCAGTA	GCCACAACATTCTCGTGAGGA
*ndhF4*	NADPH dehydrogenase - NDH-1_4_ complex	TGGCTAGATGAAGCGATGGAA	CCACCACAGTGTTTCGCAAA
*ndhD4*	NADPH dehydrogenase - NDH-1_4_ complex	TTGCGAGGTCTATTAAACCCAGAA	CCTAAAATCATGAGACTGCCAACC
*ndhL*	NADPH dehydrogenase - NDH-1_4_ complex	CCTAGACACAAACCTAATCATCCTG	GGCATAAACTATGGCTGGCATT
*chpX*	NADPH dehydrogenase - NDH-1_4_ complex	TTTGTGTAACAACGGCAGCAGT	CCAACCTTCGAAATAGGCTTGA
*atpB*	ATP synthase – β subunit	CAAGATGTATCCGTGACCTGTGAA	TTGGTTATCCCCTAGAAGTTGTTGT
*sod*	Superoxide-Dismutase	TGTTTTGGGAAATTATGGCTCC	AGCTATTTTTCCTTCAGGTTTACCC
*glcD*	Glycolate oxidase	CCCGACCCTTCCAGTCAAA	TTTCAGCAATATTCCCACCAATT
*prx*	1-Cys peroxiredoxin	TGACAAGCGTGGAGTCAAAGTC	GATTCTGCGTCATCTACACTTAGGG
*glnA*	Glutamine synthetase	AATTTGGAAAGACGGAGAGCC	AAATTAGCATAACCATCACCCCAG
*hetR*	Key regulatory gene in heterocyst differentiation	TTATATAATGGTTGAAGATACAGCTCGC	CCAGTCCTTCATTAACCGGAAA
*nifH*	Fe-protein of nitrogenase	TGGCCGTGGTATTATTACTGCTATC	GCAAATCCACCGCAAACAAC
*rbcL*	RubisCO Large subunit	ACTGCCCCTACCTGTGAAGAAA	CTCCTTAGCAAAATCTGCACGC
*rca*	RubisCO activase Small subunit	GCTTTATTTATTAATGACTTAGATGCAGGT	ACCCCCATCAAATCTACCAGC
*psaB*	Subunit of PSI	TCGGATCTGGTATGGAATTGC	CCATCGTGGGTTTCAAAGTCAT
*psaC*	Subunit of PSI	TGAGACTGCTTGCCCTACTGAC	TCAGCACCCAGATAAACCCG
*psbA*	Subunit of PSII (D1)	CAGCGGTCGCGTAATCAAT	CATTCCTAAGTTAGCGCGGTTAA

The RT-qPCR results were checked for inaccurate reactions. Single measurements with deficient primer characteristics and with bad primer efficiencies according to the LinRegPCR software were removed prior to calculations [25]. Results were reported using the comparative C_T_ method (2^−ΔΔCt^ method) which calculates the relative changes in gene expression determined from RT-qPCR experiments, according to [26]. We chose this relative quantification method as we compare not only different conditions but also results from a time course. To check if the efficiencies of the different primer pairs allow the usage of this method, we compared the mean efficiencies of all primer pairs. According to Schmittgen and Livak [27] a rough guide is that the efficiencies should be within 10% of each other. This provides us with a frame of values between 1.8 and 2.2. Our primers were within this range, with exceptions for *atpB*, *hetR* and *glnA* that yielded values of 1.6. We decided to include them into our calculations as the trends are still valid. The comparative C_T_ method examines the threshold cycle (Ct) and indicates the fractional cycle number at which the amount of amplified target reaches a fixed threshold [26]. The Ct values of the gene of interest (GOI) are normalized first to the 16S rRNA gene which is used here as the endogenous reference gene for each time point. This results in ΔCt values which are equal to the differences in thresholds for the GOI and the endogenous reference gene [26]. In time course experiments, the gene expression is often compared internally by normalization to a calibrator, which can be the time zero point. Here we have chosen the average ΔCt values of the *nifH* from the 400 µatm/25°C treatments (our control treatment, already normalized to 16S rRNA) as a calibrator, since we wanted to compare the relative abundance of the different genes, as well as their time dependence. The expression of this gene was relatively constant over the day.

The *rnpB* gene, encoding RNase P, was examined as a potential endogenous reference gene and revealed unexpected large variations in its expression. Therefore we decided to use 16S rRNA for further calculations, as its expression was stable under all the different conditions. Following Bustin et al. [28], standard deviations were chosen to present statistical differences between independent replicates of mRNA transcript enrichments.

### Statistical analysis and presentation

**Figure 1:**
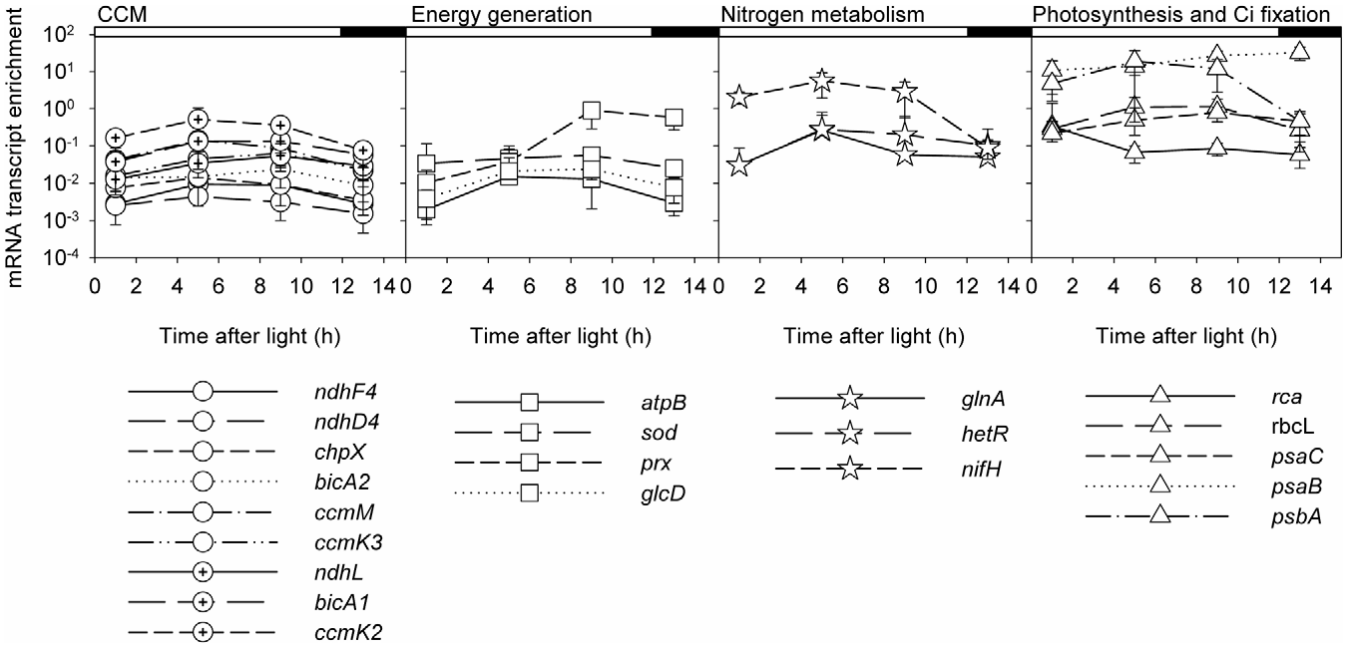
mRNA transcript enrichment of 21 GOIS from *Trichodesmium* IMS101. Culture were grown under an acclimation matrix of pCO_2_ (400 and 900 µatm) and temperature (25 and 31°C). The curves are an average of the gene expression levels under all four acclimations. The genes are arranged on the same y-axis scale from left to right, according to their functions: CCM (circles), Energy generation (rectangles), nitrogen metabolism (starts) and photosynthetic and Ci fixation (triangles). Relative abundance estimated according to the 2^−ΔΔCt^ method, with 16S rRNA as the endogenous reference gene, and average ΔCt values of the *nifH* from the 400 µatm/25°C treatments (control) as a calibrator. White and black bars on top of the graphs represent light and dark hours, respectively. n = 11–13 for each time point. Errors are ±1 standard deviation, following Bustin et al. (2009). Note that the results and standard deviations are presented using logarithmic scale y axes.

mRNA abundances of all 21 GOIs are presented in Figure 1 as the average mRNA abundance from all acclimations at each time point, n = 12–13.

**Figure 2:**
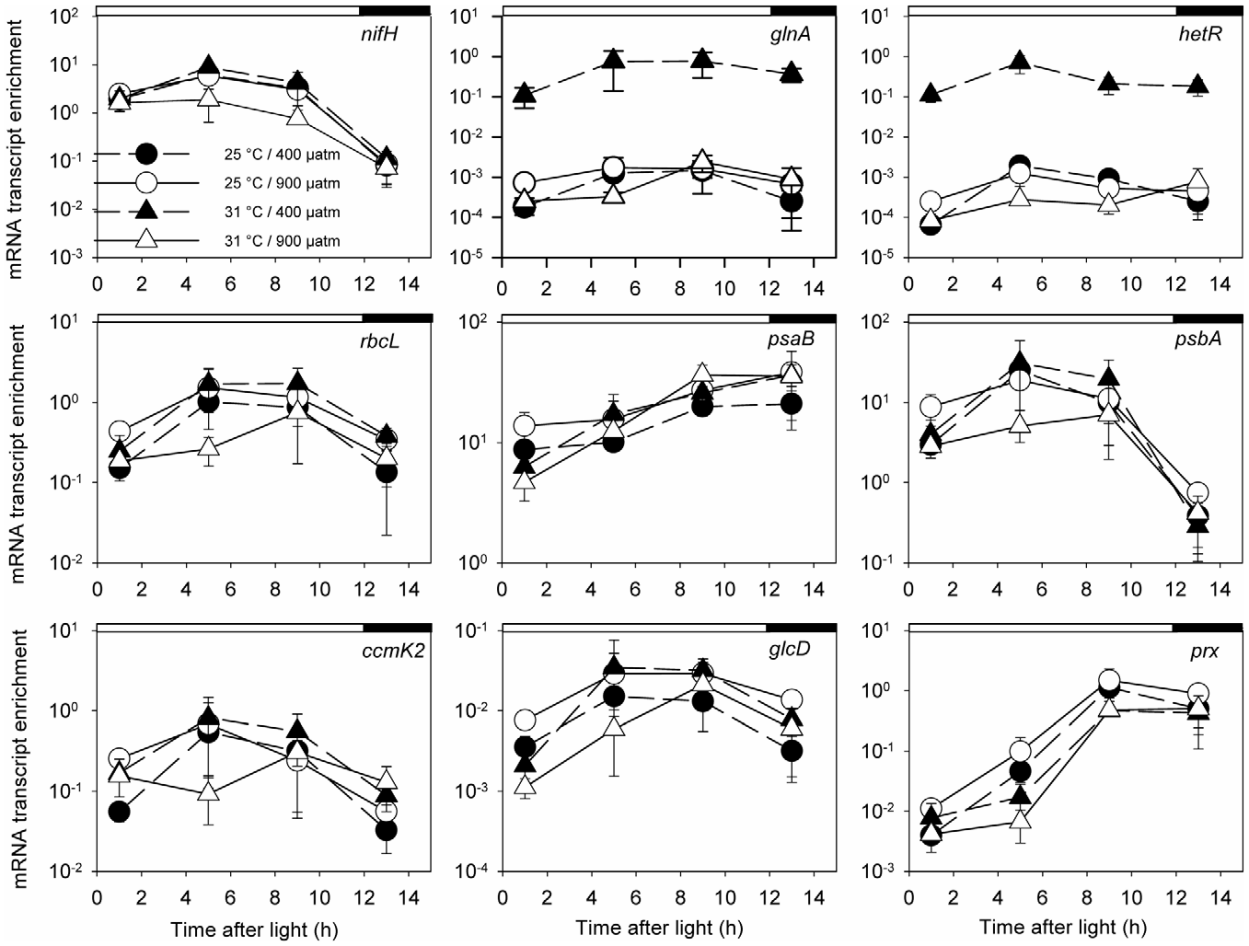
Daily mRNA transcript enrichment of the nine GOIs, significantly influenced by the changing environmental factors. Significant influence of pCO_2_ (400 and 900 µatm) temperature (25 and 31°C) and their interaction on the GOI expression was determined according to a 3-Way ANOVA (p<0.05, Table 2). All genes were also influenced by time. The upper panel presents genes related to nitrogen metabolism (*nifH, glnA* and *hetR*), the middle panel represents genes related with Ci fixation (*rbcL*) and photosynthesis (*psaB, psba*) and the lower panel represents genes related to CCM (*ccmK2*) and energy generation (*glcD* and *prx*). Circles and triangles represent *Trichodesmium* acclimated to 25°C and 31°C, respectively. Black and open symbols represent *Trichodesmium* acclimated to 400 and 900 µatm pCO_2_, respectively. Relative abundance estimated according to the 2^−ΔΔCt^ method, with 16S rRNA as the endogenous reference gene, and average ΔCt values of the *nifH* from the 400 µatm/25°C acclimation (control) as a calibrator. White and black bars on top of the graphs represent light and dark hours, respectively. n = 3 for each gene at each time point per treatment. Errors are ±1 standard deviation, following Bustin et al. (2009). Note: 1. the different y-axes scales; 2. the results and standard deviations are presented using logarithmic scale y axes.

To examine the influence of sampling time and the applied environmental factors, pCO_2_ and temperature, we performed a 3-Way ANOVA (time, pCO_2_ and temperature, p<0.05) for the enrichment values of each GOI over the time course measured. Number of independent replicates was n = 24–25 for each temperature, n = 23–25 for each pCO_2_ concentrations, and n = 12–13 for each measuring time point (Figure 2 and Table 2).

**Table 2:** The influence of changing environmental conditions on the enrichment of all our GOIs.

*Significant interactions between factors* *(additional to main factors)*				*Significant main factors*			*Time is the only influencing main factor*	*No influencing factor*
Time + pCO_2_ + temperature	pCO_2_ +time	temperature + time	pCO_2_ + temperature	Time, pCO_2_, temperature	Time, temperature	Time, pCO_2_		
nifH	glnA	prx	nifH	hetR	prx	nifH	bicA1	bicA2
glnA		glnA	ccmK2	glnA		psaB	ccmM	ndhF4
			rbcL				ccmK3	ndhD4
			glcD				ndhL	chpX
			glnA				atpB	rca
			hetR				psaC	sod
			psbA				psbA	

The environmental conditions are pCO_2_, temperature, and time. Gene expression was determined using 2^−ΔΔct^ method. Statistical analysis was made using 3-Way ANOVA (p<0.05). n = 24–25 for both temperatures, n = 23–25 for both pCO_2_, and n = 11–13 for each measuring time point.

**Figure 3:**
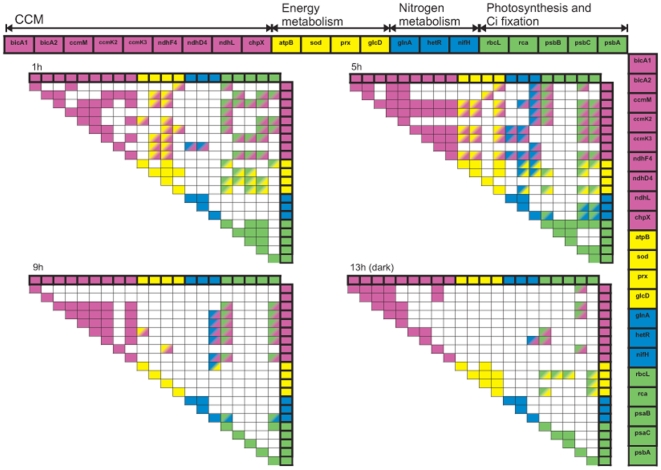
Correlations between the abundance of the 21 GOIs during the daily cycle. Sampling times were 1, 5, 9, 13 hours after the onset of light, presented from top-left to bottom-right. Colored cells represent a Pearson correlation coefficient r>0.75. Correlations are presented regardless to the different acclimations. Genes are divided to 4 groups: CCM (pink), energy metabolism (yellow), nitrogen metabolism (blue), and photosynthesis and Ci fixation (green). When correlations were between genes from the same group, the cells were colored in the groups' color. When correlations were between genes from two different groups, the cells were colored using a gradient from one groups' color to the other. All the correlation coefficients and significances according to the Pearson correlation are supplied in the supplemental data (Table S1 i-iv). Relative abundance estimated according to the 2^−ΔΔCt^ method, with 16S rRNA as the endogenous reference gene, and average ΔCt values of the *nifH* from the 400 µatm/25°C acclimation (control) as a calibrator. n = 11–13 for each gene at a given sampling time.

Pearson correlations for the enrichment values of our 21 GOIs were done for each sampling point, n = 11–13. Correlations in which the Pearson correlation coefficient- r>0.75 are presented by color coding in Figure 3 (CCM-pink, energy metabolism-yellow, nitrogen metabolism-blue, Ci fixation and photosynthesis-green). All correlation coefficients and significances according to the Pearson correlation are supplied in the supplemental data (Table S1 i-iv).

## Results and Discussion

We examined the expression levels of 21 GOIs over the day and under different pCO_2_ concentrations (400 and 900 µatm) and temperatures (25 and 31°C). The samples for the RT-qPCR analysis were taken from the exact experimental set-up described in Levitan et al. [8]. The physiological characteristics of *Trichodesmium* IMS101 cultures used in these experiments are summarized in Table 3: growth rates (chl d^−1^), elemental stoichiometry [C∶N, C∶P, N∶P (mol∶mol)], nitrogen fixation rates (nmol N_2_ chl^−1^ h^−1^), and NifH amounts (pmol µg protein^−1^). These data were presented and discussed in Levitan et al. [8].

**Table 3:** Physiological characteristics of *Trichodesmium* IMS101 cultures acclimated to a matrix of pCO_2_ and temperature.

	400 µatm pCO_2_/25°C	400 µatm pCO_2_/31°C	900 µatm pCO_2_/25°C	900 µatm pCO_2_/31°C
Growth rate (chl d^−1^)^a^	0.17±0.04	0.27±0.01	0.33±0.03	0.38±0.08
C∶N (mol∶mol)^b^	7.3±0.9	6.6±0.7	6.5±0.4	5.97±0.51
C∶P (mol∶mol)^b^	139±28	164±48	118±32	196±27
N∶P (mol∶mol)^b^	19.5±5.4	25.2±9.0	18.3±5.5	33.1±4.9
Maximal N_2_ fixation rate (nmol N_2_ chl^−1^ h^−1^)^a^	4.8(1.4	7.2(3.1	20(5.3	15.6(3.2
NifH (pmol (g protein-1) at the time of maximum N2 fixationa	0.26±0.05	0.3±0.06	0.27±0.03	0.28±0.09
GlnA (pmol µg protein^−1^) at the time of maximum N_2_ fixation^a^	0.05±0.01	0.05±0.02	0.07±0.02	0.07±0.02

a - n = 3–4

b - n = 12–13

Acclimation pCO_2_ levels were 400 and 900 µatm and temperatures were 25 and 31°C. The extended data set and discussion of the physiological responses were previously reported (Levitan et al., 2010a) and are summarized here to emphasize physiological changes associated with the gene abundance presented from the same cultures.

The GOIs can be divided to 4 functional groups: CCM (*bicA1, bicA2, ccmM, ccmk2, ccmK3, ndhF4, ndhD4, ndhL, chpX*), energy metabolism (*atpB, sod, prx, glcD*), nitrogen metabolism (*glnA, hetR, nifH*), and photosynthesis and Ci fixation (*rbcL, rca, psaB, psaC, psbA*). The gene description and the primers sequences are presented in Table 1.

### Expression levels of the selected GOIs

We examined the mean enrichment levels of the different GOIs over the day, arranged by their metabolic function, using the average enrichment values of all acclimations (Figure 1). The expression levels of each of our GOIs were on the same order of magnitude for all acclimations (excluding the high pCO_2_/high temperature acclimation for *hetR* and *glnA*; Figure 1).

The transcript abundance of the CCM and energy metabolism GOIs were low compared to the nitrogen metabolism and Ci fixation and photosynthetic genes. The CCM and energy metabolism genes spanned over 2 orders of magnitude, with the exception of *ccmK2* and *prx* (Figure 1). The *prx* gene, encoding for the cyanobacterial peroxiredoxin 1-Cys revealed a unique diurnal trend and had higher transcript abundance relative to the other genes examined for energy metabolism (Figure 1). The *ccmK2* gene is encoding for the main β-Carboxysome shell protein. The pores in the CcmK2 protein hexamers may enable diffusion of small essential metabolites into the carboxysome lumen [23].The *ccmK2* gene revealed a trend similar to the other CCM genes, yet its expression was slightly higher (Figure 1). Most GOIs related to nitrogen metabolism (*glnA, hetR* and *nifH*) and photosynthesis and Ci fixation (*rbcL, rca, psaB, psaC, psbA*), exhibited 1–4 orders of magnitude higher average enrichment levels than the CCM and energy metabolism genes with varying expression patterns (Figure 1). Our findings correspond with results published from a community gene expression of a *Trichodesmium* spp. bloom in the Southwest Pacific Ocean showing that during the day, the highest abundance of *Trichodesmium* related genes was that of the photosynthetic and nitrogen metabolism pathways [29].

We previously showed the pCO_2_ influences gene expression patterns over the diurnal cycle for five of the above GOIs, *nifH, hetR, glnA, psaB* and *psbA*, in *Trichodesmium* IMS101 (data from another set of experiments, [5]). Three of these genes, *nifH, psaB* and *psbA,* had similar enrichment levels in both studies (Figure 1, [5]). The combination of elevated temperature and high pCO_2_, (not examined in [5]) significantly increased the transcript abundance of *glnA* and *hetR*. This acclimation resulted in ∼2 orders of magnitude higher transcript levels than previously reported for *glnA* (Figure 2, [5]). The increase in *glnA* transcript abundance was not reflected in the GlnA protein pool size, and there was no significant difference for the GlnA amount between treatments and over the diurnal cycle (as measured by a quantitative western blot, one way ANOVA, p<0.05; Table 3; [5]). The combined influence of elevated pCO_2_ and high temperature increased the average *hetR* enrichment levels to levels similar to those previously reported [5]. In *Trichodesmium*, the *hetR* gene was suggested to be constitutively expressed (under a 12∶12 Light/Dark cycle), yet it's diurnal abundance ranged 3–10 fold and was regulated by combined nitrogen concentration levels [30]. Based on our results, we believe that the sensitivity of *hetR* to changes in pCO_2_, temperature and time, further points to an environmental sensitivity of this gene.

### The influence of CO_2_ and temperature on the selected GOIs

To determine whether the diurnal cycle interacted with the applied environmental factors to influence the transcript abundance of the GOIs, we applied a 3-Way ANOVA (pCO_2_, temperature and time of day) for all the GOIs tested. The results are summarized in Table 2 and the enrichment levels of the influenced genes for all acclimations are presented in Figure 2. Out of 21 GOIs, six genes (*bicA1, ccmM, ccmK3, ndhL, atpB* and *psaC*; Table 2) were influenced by the diel cycle alone and six other genes (*bicA2, ndhF4, ndhD4, chpX, rca* and *sod*) were influenced by neither time nor the applied environmental factors. Transcripts abundances of GOIs that were not affected by any of the three main factors, or were influenced by time only, are presented in the supplemental data (Figure S1). Only nine GOIs were significantly affected by pCO_2_ and/or temperature (*nifH, glnA, hetR, rbcL, psbA, psbA prx, glcD,* and *ccmK2*). Seven of the nine GOIs that appeared sensitive to pCO_2_ and/or temperature (*nifH, glnA, hetR, rbcL, psbA, psbA,* and *prx*; Figure 2) correspond with the nine genes expressed at the highest abundance (Figure 1). These genes are representative of the photosynthetic and Ci fixation, nitrogen metabolism and energy generation pathways.

While photosynthesis in *Trichodesmium* is relatively insensitive to changes in pCO_2_ [4], [6], N_2_ fixation rates vary significantly with changes in ambient pCO_2_ [3]–[5], [8]. N_2_ fixation, and possibly the sequential assimilation of ammonium, were affected by pCO_2_ at the mRNA and activity level, while protein pools remained relatively constant (Table 3; [5]). Abundance of the nitrogenase Fe-protein gene, *nifH,* was affected by pCO_2_, time of day, and the combined influence of pCO_2_ and temperature (Figure 2; [5]). This corroborates findings showing that *nifH* expression is pCO_2_ sensitive [5] and controlled by a circadian rhythm [31]. Relative stability of *nifH* expression to temperature changes was also reported for *Trichodesmium* IMS101 grown at 24, 28.5 and 31°C [31]. Similarly, temperature did not appreciably affect the abundance of the NifH protein and the nitrogenase N_2_ fixation rates in the temperature range applied here (Table 3; [5]). *Trichodesmium* cultures tested under a broader temperature range revealed changes in growth and N_2_ fixation rates [32]. As natural populations of *Trichodesmium* spp. range from 20 to 34°C (reviewed in [33]), it would be advisable to further examine the acclimation responses and levels of regulation under a wider temperature range.

*glnA* and *hetR* transcripts were statistically influenced by all three variables: pCO_2_, temperature and time of day (Table 2), in line with the reported influence of pCO_2_ and time on both genes [5], [30], [34]. To our knowledge, scant data on the effect of environmental conditions on *glnA* and *hetR* expression in *Trichodesmium* shows that fixed-nitrogen sources and diurnal rhythmicity regulated *glnA* expression in natural populations of marine *Synechococcus* spp [35] and *hetR* expression in *Trichodesmium* [30]. The *hetR* gene, previously suggested to be involved only in heterocysts differentiation, was also found in the non-heterocystous filamentous diazotroph *Symploca* PCC8002 [36] and *Lyngbya* PCC8106 [37]. The existence of *hetR* in *Trichodesmium*, *Symploxa* PCC8002a and *Lyngbya* PCC8106, its regulation by combined nitrogen status and time [36], [37], and the apparent sensitivity of *Trichodesmium's hetR* to pCO_2_ (also affecting N_2_ fixation; Figure 2, Table 2), further suggest that *hetR* must play a critical role in diazotroph nitrogen metabolism and is not limited to heterocyst differentiation [37].

The regulation of photosynthetic genes is essential in *Trichodesmium* where photosynthetic O_2_ evolution is separated from N_2_ fixation by a complex spatial-temporal strategy. To enable N_2_ fixation, down regulation of photosystem II (PSII), possibly controlled by redox state of the plastoquinone (PQ) pool, occurs at midday [13]. Changing expression levels and patterns of photosynthetic genes can influence *Trichodesmium's* metabolism. In *Trichodesmium*, the expression pattern of *psa*B and *psbA* were affected by pCO_2_ [5]. Our data reveal that while photosystem I (PSI) core protein gene *psaC* was influenced only by time, *psaB* (PSI) was influenced by both pCO_2_ and time and *psbA* (PSII) was influenced by time and the interaction between pCO_2_ and temperature, with no apparent sensitivity to temperature as the predominant factor (Table 2, Figure 2). *psaA* and *psbA* are controlled by a circadian rhythm and their expression pattern did not change between 24 and 28.5°C [12]. *psaA* and *psaB* are closely located in *Trichodesmium's* genome and their interaction is highly conserved for many cyanobacteria [38]. Therefore, we deduce that the temperature insensitivity of *psaA* [12] corroborates our finding (Table 2, Figure 2).

Three additional genes, *ccmK2*, *rbcL* and *glcD,* were influenced by the 2-Way interaction of pCO_2_ and temperature (Table 2). Presently, there is no report on the expression and regulation of these genes in *Trichodesmium*. In *Synechococcus* PCC7942, but not in all cyanobacteria, the *ccm* operon is located in the 5′-flanking region on the *rbcL-rbcS* operon [39], [40]. However, our genomic analysis reveals that *rbc*L and *ccmk2* are not closely oriented in the *Trichodesmium* genome. In *Synechocystis* PCC6803, *rbcL* expression was insensitive to changes in pCO_2_ (0 to 3% CO_2_ in air; [41]). Our findings show that *rbcL* expression was modified only when combining high pCO_2_ with high temperature, yet its abundance was still at the same order of magnitude for all acclimations. In *Synechocystis* PCC6803 the Ci derived transcriptional changes in *rbcL* transcript amount were uncoupled from changes in RbcL protein level, possibly resulting from low protein turnover rate due to the protective effect of the carboxysome, slowing down the protein degradation [42]. This was also indicated for *Trichodesmium* in our study. While pCO_2_ alone and the combination of pCO_2_ and temperature influenced the RbcL protein amount, the highest protein level was at 900 µatm/25°C while the highest transcript level appeared at 900 µatm/31°C (unpublished data; Figure 2).

The oxygenase activity of RubisCO forms 2-phosphoglycolate (2PG), considered toxic for Ci fixation in the Calvin cycle. The GlcD protein helps protect the Calvin cycle by converting two molecules of 2PG into one 3-phosphoglycarate (3PGA) molecule (the product of RubisCO's carboxylase activity), and thus enables Ci fixation to proceed. *glcD* metabolism was found essential for the viability of the cells and oxygenic photosynthesis in the cyanobacterium *Synechocystis* PCC6803 at ambient CO_2_ conditions [43]. Statistical analysis (3-Way ANOVA, p<0.05) revealed that the *glcD* mRNA abundance was sensitive to the combined influence of pCO_2_ and temperature, yet its abundance was the same for all our acclimations (Figure 2, Table 2). Although *glcD* is also found in *Arabidopsis* and *Anabaena* [43], there is generally scarce information regarding the expression and regulation of this gene.

Temperature, time of day and their interaction affected the 1-cys peroxiredoxin gene, *prx* (Table 2), increasing its abundance by 3 orders of magnitude from 1 to 9 h after the onset of light (Figure 2). 1-cys *prx* mRNA increased in response to different metabolic imbalances in *Synechocystis* PCC6803, including irradiation, salinity, and iron deficiency [44]. No data are currently available on changes of *prx* at different pCO_2_ and/or temperatures in *Trichodesmium* and other cyanobacteria. O_2_ generated in PSII is reduced to H_2_O_2_ by PSI related components [45]. In cyanobacteria peroxiredoxin reduces H_2_O_2_ to H_2_O using electrons donated from a variety of substrates [46]. Increased expression of iron and oxidative stress genes at the end of the high N_2_ fixation period was detected for cultures of the unicellular diazotroph *Crocosphaera watsonii* [47]. Biological fixation of one N_2_ molecule requires at least 16 ATP molecules that can be generated via cyclic electron flow around PSI [48]. Thus, in *Trichodesmium*, the higher expression of *prx* in the second half of the photoperiod may be required to recover from the high energetic demand for N_2_ fixation, leaving the cell susceptible to oxidative stress. In addition, peroxidases function as regulators of redox-mediated signal transduction in some eukaryotes [49], [50], and are therefore important components for the cellular antioxidant defense system and redox homeostasis [46]. Redox state of shared components between photosynthesis and respiration regulates gene expression in *Trichodesmium* [13], [51]. Hence, changes in *prx* expression reported here (Figure 2, Table 2) indicate that oxidative defense, photosynthesis and/or respiratory redox state in *Trichodesmium* are temperature and time dependent.

Our results indicate that these nine genes (*nifH, glnA, hetR, rbcL, psbA, psaB prx, glcD* and *ccmK2*) are non-constitutively expressed and are regulated both by a diurnal cycle and by environmental factors such as pCO_2_ and temperatures.

### Expression of CCM genes

The nine CCM-related GOIs that were tested are representative of all known CCM complexes in *Trichodesmium:* the carboxysome (*ccmM, ccmK2, ccmK3*) that contains the cellular Ci fixation enzyme RubisCO (*rbcL*; [19]), HCO_3_^−^ transporter named BicA (*bicA1*, *bicA2*), and the specialized NADPH dehydrogenase NDH-I_4_ (*ndhF4*, *ndhD4*, *ndhL*, and chpX). *Trichodesmium* lacks any genes of inducible-high affinity uptake system for both CO_2_ and HCO_3_^−^, such as NDH-I_3_ (CO_2_), BCT1 or SbtA (HCO_3_^−^; [18], [19]), and has no recognizable carbonic anhydrase (CA) genes [18].

CCM operation in algae is regulated by environmental factors with elevated CO_2_ levels expected to reduce the cellular requirements for concentrating Ci, and enabling enhanced growth [52]. All of our nine examined CCM-related GOIs (Table 1) exhibited similar expression patterns and low expression levels when compared to Ci fixation, photosynthesis and nitrogen metabolism genes (Figure 1). Only one gene, the *ccmK2*, was affected by changes in environmental conditions (Figure 2, Table 2). For all time points measured, the expression of the CCM related genes had the highest correlations of all the GOIs metabolic groups. This applies within the CCM group and also with the other functional groups (Figure 3).

CCM genes of high Ci affinity are known to be regulated at the transcript level [53]. Our experimental setup is different in two aspects from “classical” cyanobacterial CCM induction experiments: 1. we report on a steady state expression of CCM genes under long term constant CO_2_ conditions, whereas in most publications cells are rapidly transferred (usually less then 1 day) from one CO_2_ concentration to another; 2. Trying to work on ecologically relevant pCO_2_ concentrations, we applied 400 and 900 µatm pCO_2_. This change is very small in comparison to concentrations >1% CO_2_ that are usually referred to as high CO_2_ in CCM-literature. As publications reporting steady state CCM gene expression and acclimations to ecologically relevant pCO_2_ levels are scarce we will use the available literature to discuss our data.

In *Synechocystis* PCC6803, genes of low affinity CCM components such as *ndhD4, ndhF4. chpX,*and *ccmK-N* [41], [42] and *bicA* [42], [53] were Ci insensitive, constitutively expressed, and revealed relatively low transcript abundance as we found for *Trichodesmium* (Figures 1 and 3, Table 2). In *Synechococcus* PCC7002 *bicA* is regulated by a *ccmR* gene [53], which is absent in the *Trichodesmium* genome [19].

In *Trichodesmium's* genome, most of the CCM-related genes are not arranged in operons or clusters (http://genome.jgi-psf.org/finished_microbes/trier/trier.home.html), as was previously shown for *Synechocystis* PCC6803 [54]. This also applies for the three CCM-related gene-pairs that were co-expressed over the day, *ccmM*-*ccmK3*, *ccmK3*-*ccmK2* (caboxysome shell) and *ndhF*4-*ndhD4* (NDH-I_4_; Table 4). We conclude that in *Trichodesmium*, CCM genes are constitutively expressed and are mostly unaffected by the applied changes in pCO_2_ and temperature.

**Table 4:** Correlations between four GOI pairs that were co-expressed at all measured time points.

*Hours after the onset of light*				*Gene pair*
*13 h (dark)*	*9 h*	*5 h*	*1 h*	
0.767 (0.004)	0.781 (0.003)	0.802 (0.000)	0.828 (0.002)	*ccmM-ccmK3*
0.792 (0.002)	0.816 (0.001)	0.911 (0.000)	0.778 (0.005)	*ccmK3-ccmK2*
0.913 (0.000)	0.923 (0.000)	0.963 (0.000)	0.832 (0.001)	*ndhF4-ndhD4*
0.912 (0.000)	0.990 (0.000)	0.969 (0.000)	0.978 (0.000)	*hetR-glnA*

Presented values are the Pearson correlation coefficient and the significance is in the parentheses. Sampling points were 1, 5, 9, 13 hours after the onset of light.

Genomic analyses indicate that *Trichodesmium* lacks inducible inorganic carbon (Ci) uptake systems [18], [19]. Yet, physiological measurements of Ci uptake showed that *Trichodesmium* changes its Ci uptake characteristics when acclimated to high CO_2_ (900 µatm; [6], [7]). While the cell's affinity to total DIC decreased with elevated pCO_2_ [6], the cell's CO_2_ uptake increased [7]. Under a range of pCO_2_ (150-900 µatm pCO_2_), *Trichodesmium* uses HCO_3_^−^ for over 90% of its Ci source ([6]; Kranz and Levitan, unpublished data). Based on the genetic analysis (Figure 1; [19]), the Km of Ci uptake [6] and BicA being a low-affinity but high flux HCO_3_^−^ uptake system [20], it is likely that a major part of *Trichodesmium's* Ci uptake is via the Na^+^ dependent HCO_3_^−^ transporter BicA.

The operation of a Ci uptake system that maintains constant transcription levels while its affinity is modified indicates that CCM operation in *Trichodesmium* is controlled at the translational or post-translational levels. Changes in CCM operation without altering gene expression or the cell's capacity to transport Ci was proposed by Beardall and Giordano [52], i.e. via fluctuations in the redox state of the PQ pool. A rapid increase in HCO_3_^−^ transport activity appears to involve phosphorylation events, possibly by activating two or three component regulatory systems, is of considerable importance when looking at CCM regulation [55], [56]. The thioredoxin regulatory system and internal Ci pools can also act in controlling CCM operation, away from the transcript level [55], [57]. Moreover, it is possible that large transcript changes were not detected in *Trichodesmium's* CCM genes due to the long acclimations (>2 months) of the cultures, whereas a rapid transfer of *Trichodesmium* from low to high CO_2_ may result in changes in transcript abundance. Finally, although there are physiological changes in cells grown at different pCO_2_s [2]–[8], it could be that the acclimation to 900 µatm pCO_2_ does not simulate a large enough increase to detect significant differences in CCM gene abundance.

Our analyses show low transcript abundance of co-expressed CCM genes in *Trichodesmium* (Figures 1 and 3) that are insensitive to changes in pCO_2_ and temperature (Figure 2; Table 2). This, together with genomic analysis [19] and physiological data, suggest that CCM genes in *Trichodesmium* are constitutively expressed under our applied conditions.

### Co-expression of GOIs

We explored co-expression of GOIs by examining the correlations between their enrichment at all measured time points. Figure 3 presents GOIs with Pearson correlation coefficients higher than 0.75 (r>0.75, p<0.01, all correlations are given in Table S1 i-iv in the supplemental data). The highest number of significant correlations between GOIs of different metabolic functions appeared 5 h after the onset of light, when high N_2_ fixation rates and assimilation are detected [5], [13], [58]. A large number of correlations were also observed between genes 1 h after the onset of light. Later in the day, at 9 and 13 h, a significantly lower number of correlations were detected, especially between GOIs related to different metabolic groups, indicating only limited co-expression.

Expression levels of nitrogen metabolism GOIs (*glnA*, *hetR*, *nifH*) were correlated with GOIs of other metabolic functions predominantly at 5 and 9 hours after the onset of light, yet the highest number of correlations was found at 5 h (Figure 3). The limited literature on gene expression patterns in *Trichodesmium* demonstrated diurnal regulation of genes correlated with photosynthesis and nitrogen metabolism [12], [30], [31], [35], [59]. At 9 h, only *nifH* was co-expressed with GOIs of other metabolic functional groups, especially with the carboxysomal, NDH-I_4_ and photosynthesis related genes. Chen et al. [12] demonstrated a time-dependent cycling and coupling between *nifH* and photosynthetic transcripts in *Trichodesmium*. Our results showed that *nifH* was co-expressed with the photosynthesis-related genes (*psaC*, *psbA* and *rbcL*), with all CCM components, and with two of the energy metabolism genes (*atpB* and *sod*).

Out of the three nitrogen metabolism genes tested, only the *glnA*-*hetR* gene pair was co-expressed for all time points measured (Figure 3, Table 4), although they are not closely localized in the *Trichodesmium* genome (http://genome.jgi-psf.org/finished_microbes/trier/trier.home.html). *hetR* was also highly correlated to the NDH-I_4_ genes, to *atpB*, and to *sod*. The co-expression of *glnA* and *hetR* did not correspond with *nifH* expression (Figure 3), in agreement with other studies showing that *hetR* expression was inversely correlated with *nifH* expression in *Trichodesmium* [30], [34]. In *Trichodesmium*, both *glnA* and *hetR* are likely under *ntcA* regulation [34], yet we couldn't verify this in our experiment.

Energy metabolism related GOIs (*atpB*, *sod*, *prx*, and *glcD*) were co-expressed and positively correlated with all CCM related GOIs and photosynthetic genes at 1 and 5 h after the onset of light. This could be related to the energetically demands of the CCM, and to the connection between photosynthetic electron transfer and the use of these electrons in sequential processes (Figure 3; [6], [7]). *prx* correlated with many other GOIs only 1 h after the onset of light. This correlation disappeared from 5 h onwards, when *prx* mRNA abundance rapidly increased (Figures 1 and 2).

Positive correlations between Ci fixation and photosynthesis to CCM GOIs were observed at all measured time points. A high number of correlations were especially noted 1 and 5 h after the onset of light for *rbcL* and *psbA* together with the CCM GOIs (i.e. *rbcL-ccmK2* at 1, 5 and 9 h). Fewer correlations, predominantly occurring at 5 h, were detected between photosynthetic and nitrogen metabolism GOIs (Figure 3). Co-expression of CCM and photosynthetic genes at the first half of the photoperiod (when photosynthesis and carbon fixation take place) could account for the tight interaction observed between the two mechanisms [6], [20].

The co-expression of GOIs we observed fundamentally reflects the diurnal patterns of the predominant metabolic pathways in *Trichodesmium* (CCM, photosynthtesis and carbon fixation, nitrogen metabolism, and energy generation). Transcriptional regulation is the first level of regulation, followed by translational and post-translational regulation. Different levels of metabolic regulations were found in *Trichodesmium*, for example for nitrogenase [5], [16], [17], [31] and PSII [8], [12]. The differing patterns of co-expression between the metabolic gene families during the day (Figure 3) indicate a strategy of a complex and tightly regulated gene expression. In *Trichodesmium*, such a strategy is required due to the unique spatial-temporal segregation of oxygenic photosynthesis and N_2_ fixation [12]–[14].

### Conclusions

Our motivation in this study was to examine changes in expression of essential metabolic genes in *Trichodesmium* grown under a matrix of pCO_2_ and temperature. In *Trichodesmium* IMS101, nitrogen metabolism, Ci fixation, and photosynthesis related GOIs exhibited the highest abundance of all measured genes (Figure 1). These genes were also mostly affected by changes in pCO_2_, temperature and the time within the diurnal period (Figure 2, Table 2), suggesting that these metabolic functions are also controlled at the mRNA transcript level. To our knowledge this is the first report of CCM gene expression in *Trichodesmium*. We suggest that CCM genes in *Trichodesmium* are constitutively expressed under our applied conditions, yet, their corresponding protein activity may be altered by changes in pCO_2_ [6], [7], probably due to translational and/or post-translational regulations [19].

Protein and activity levels of the CCM and fixation pathways in *Trichodesmium* are influenced by environmental changes [5]–[8]. Thus, we hypothesized that modifications in the CCM genes expression due to elevated pCO_2_ may facilitate the reported physiological changes. Our results negate this hypothesis as the expression of CCM-genes under long term acclimation (steady state conditions) was insensitive to changes in experimental conditions. The comprehensive analysis of abundance and expression patterns of the GOIs presented here, demonstrates that gene expression may be uncoupled from translational and protein activity levels. Thus, although gene expression reflects active metabolic pathways, there is often an uncoupling between transcription and enzyme activity. Therefore we conclude that to examine the effects of environmental parameters on *Trichodesmium* and its biogeochemical impact, studies of gene transcript levels should by be done in parallel with physiological and activity measurements.

## Supporting Information

Figure S1 **Daily mRNA transcript enrichment of 12 GOIs, not significantly influenced by changing environmental factors**. Significant influence of pCO_2_ (400 and 900 µatm) temperature (25 and 31 °C) and their interaction, on the GOI expression was done according to a 3‐Way ANOVA (p<0.05, Table 3). The left panel represent GOIs that no influencing factor (*bicA2*, *ndhD4*, *ndhF4*, *chpX*, *rca, sod*) and the right panel represent genes for which time was the only influencing factor. Circles and triangles represent *Trichodesmium* acclimated to 25 °C and 31 °C, respectively. Black and open symbols represent *Trichodesmium* acclimated to 400 and 900 µatm pCO_2_, respectively. Relative abundance estimated according to the 2^‐ΔΔCt^ method, with 16S rRNA as the endogenous reference gene, and average ΔCt values of the *nifH* from the 400 µatm / 25 °C acclimation (control) as a calibrator. White and black bars on top of the graphs represent light and dark hours, respectively. n = 3 for all. Errors are ±1 standard deviation, following Bustin et al. (2009). Note: 1. the different y‐axes scales; 2. the results and standard deviations are presented using logarithmic scale y axes.
(TIF)

Table S1 **Pearson correlations of the enrichment of the 21 genes of interest over the day.** i‐ 1 h after the onset of light; ii‐ 5h after the onset of light; iii‐ 9 h after the onset of light; iv‐ 13 h after the onset of light. Relative abundance estimated according to the 2^‐ΔΔCt^ method, with 16S rRNA as the endogenous reference gene, and average ΔCt values of the nifH from the 400 µatm / 25°C treatments as a calibrator. n = 11‐13 for each gene at a given sampling time. Upper value represents the correlation coefficient (r) and lower values represent the significance (p).
(DOCX)
