# Supplementary figures and images for: The Influence of pCO2 and Temperature on Gene Expression of Carbon and Nitrogen Pathways in Trichodesmium IMS101
Source: PLoS One. 2010 Dec 6;5(12):e15104. doi: 10.1371/journal.pone.0015104 (PMC2997788; doi:10.1371/journal.pone.0015104)

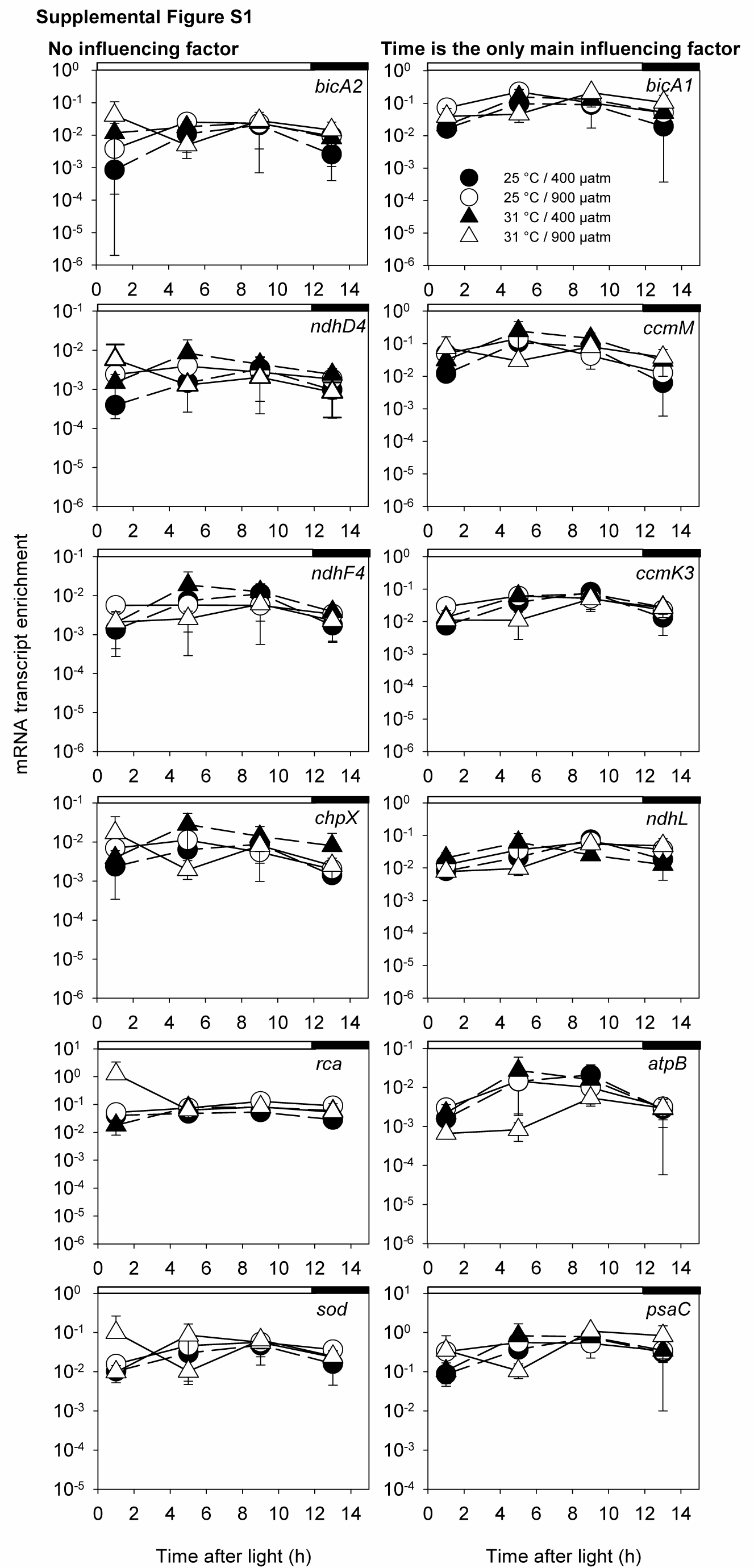

Supplement: Figure S1 — Daily mRNA transcript enrichment of 12 GOIs, not significantly influenced by changing environmental factors. Significant influence of pCO2 (400 and 900 µatm) temperature (25 and 31 °C) and their interaction, on the GOI expression was done according to a 3‐Way ANOVA (p<0.05, Table 3). The left panel represent GOIs that no influencing factor (bicA2, ndhD4, ndhF4, chpX, rca, sod) and the right panel represent genes for which time was the only influencing factor. Circles and triangles represent Trichodesmium acclimated to 25 °C and 31 °C, respectively. Black and open symbols represent Trichodesmium acclimated to 400 and 900 µatm pCO2, respectively. Relative abundance estimated according to the 2‐ΔΔCt method, with 16S rRNA as the endogenous reference gene, and average ΔCt values of the nifH from the 400 µatm / 25 °C acclimation (control) as a calibrator. White and black bars on top of the graphs represent light and dark hours, respectively. n = 3 for all. Errors are ±1 standard deviation, following Bustin et al. (2009). Note: 1. the different y‐axes scales; 2. the results and standard deviations are presented using logarithmic scale y axes. (TIF) [file pone.0015104.s001.tif]
