# Supplementary material for: The Influence of pCO2 and Temperature on Gene Expression of Carbon and Nitrogen Pathways in Trichodesmium IMS101
Source: PLoS One. 2010 Dec 6;5(12):e15104. doi: 10.1371/journal.pone.0015104 (PMC2997788; doi:10.1371/journal.pone.0015104)
Supplement: Table S1 — Pearson correlations of the enrichment of the 21 genes of interest over the day. i‐ 1 h after the onset of light; ii‐ 5h after the onset of light; iii‐ 9 h after the onset of light; iv‐ 13 h after the onset of light. Relative abundance estimated according to the 2‐ΔΔCt method, with 16S rRNA as the endogenous reference gene, and average ΔCt values of the nifH from the 400 µatm / 25°C treatments as a calibrator. n = 11‐13 for each gene at a given sampling time. Upper value represents the correlation coefficient (r) and lower values represent the significance (p). (DOCX) [file pone.0015104.s002.docx]

**Table S1 i-iv -**

S1i-

| *bicA1* | *bicA2* | *ccmM* | *ccmK2* | *ccmK3* | *ndhF4* | *ndhD4* | *ndhL* | *chpX* | *atpB* | *sod* | *asp* | *glcD* | *glnA* | *hetR* | *nifH* | *rbcL* | *rca* | *psaB* | *psaC* | *psbA* |  |
| --- | --- | --- | --- | --- | --- | --- | --- | --- | --- | --- | --- | --- | --- | --- | --- | --- | --- | --- | --- | --- | --- |
| 1 | 0.349  0.266 | 0.595  0.041 | 0.785  0.002 | 0.627  0.039 | 0.707  0.015 | 0.514  0.088 | 0.093  0.786 | 0.4159  0.133 | 0.291  0.385 | 0.408  0.188 | 0.529  0.077 | 0.824  0.002 | -0.219  0.517 | -0.186  0.584 | 0.22  0.946 | 0.811  0.001 | 0.383  0.219 | 0.707  0.010 | 0.682  0.015 | 0.312  0.323 | *bicA1* |
|  | 1 | 0.911  0.000 | 0.170  0.596 | 0.178  0.600 | -0.152  0.655 | 0.966  0.000 | 0.768  0.006 | 0.979  0.000 | 0.202  0.551 | 0.981  0.000 | 0.925  0.000 | -0.064  0.853 | 0.677  0.22 | 0.558  0.074 | -0.006  0.986 | 0.593  0.042 | 0.977  0.000 | 0.832  0.001 | 0.870  0.000 | -0.117  0.717 | *bicA2* |
|  |  | 1 | 0.514  0.088 | 0.828  0.002 | 0.767  0.006 | 0.978  0.000 | 0.570  0.067 | 0.944  0.000 | 0.618  0.043 | 0.930  0.000 | 0.973  0.000 | 0.596  0.053 | 0.187  0.581 | 0.278  0.407 | 0.065  0.840 | 0.821  0.001 | 0.920  0.000 | 0.918  0.000 | 0.959  0.000 | 0.127  0.694 | *ccmM* |
|  |  |  | 1 | 0.778  0.005 | 0.800  0.003 | 0.358  0.253 | 0.431  0.188 | 0.249  0.436 | 0.498  0.119 | 0.186  0.563 | 0.410  0.185 | 0.569  0.068 | 0.163  0.632 | 0.238  0.480 | 0.176  0.584 | 0.806  0.002 | 0.163  0.612 | 0.429  0.164 | 0.491  0.105 | 0.514  0.087 | *ccmK2* |
|  |  |  |  | 1 | 0.894  0.000 | 0.856  0.001 | 0.376  0.254 | 0.805  0.003 | 0.793  0.004 | 0.650  0.031 | 0.802  0.003 | 0.733  0.010 | -0.011  0.973 | 0.045  0.896 | 0.533  0.037 | 0.9361  0.000 | 0.264  0.433 | 0.600  0.051 | 0.910  0.000 | 0.905  0.000 | *ccmK3* |
|  |  |  |  |  | 1 | 0.832  0.001 | 0.118  0.729 | 0.616  0.044 | 0.652  0.030 | 0.583  0.060 | 0.831  0.002 | 0.754  0.007 | -0.162  0.634 | -0.078  0.820 | 0.450  0.165 | 0.912  0.000 | 0.466  0.149 | 0.736  0.010 | 0.880  0.000 | 0.779  0.005 | *ndhF4* |
|  |  |  |  |  |  | 1 | 0.544  0.084 | 0.985  0.000 | 0.606  0.048 | 0.983  0.000 | 0.982  0.000 | 0.639  0.034 | 0.203  0.550 | 0.264  0.432 | 0.000  1.000 | 0.736  0.006 | 0.978  0.000 | 0.921  0.000 | 0.950  0.000 | 0.006  0.985 | *ndhD4* |
|  |  |  |  |  |  |  | 1 | 0.350  0.291 | 0.526  0.097 | 0.451  0.164 | 0.404  0.217 | 0.105  0.758 | 0.783  0.004 | 0.780  0.005 | 0.422  0.192 | 0.0416  0.204 | -0.099  0.773 | 0.049  0.886 | 0.221  0.514 | 0.240  0.477 | *ndhL* |
|  |  |  |  |  |  |  |  | 1 | 0.579  0.062 | 0.989  0.000 | 0.961  0.000 | 0.649  0.031 | 0.071  0.835 | 0.040  0.908 | 0.0074  0.982 | 0.680  0.15 | 0.983  0.000 | 0.901  0.000 | 0.937  0.000 | -0.004  0.991 | *chpX* |
|  |  |  |  |  |  |  |  |  | 1 | 0.570  0.067 | 0.920  0.002 | 0.569  0.068 | 0.270  0.422 | 0.350  0.291 | 0.663  0.026 | 0.754  0.007 | 0.179  0.599 | 0.511  0.108 | 0.709  0.014 | 0.872  0.000 | *atpB* |
|  |  |  |  |  |  |  |  |  |  | 1 | 0.955  0.000 | 0.872  0.000 | 0.026  0.94 | 0.040  0.908 | -0.058  0.857 | 0.617  0.033 | 0.999  0.000 | 0.897  0.000 | 0.897  0.000 | 0.908  0.000 | *sod* |
|  |  |  |  |  |  |  |  |  |  |  | 1 | 0.890  0.056 | 0.254  0.452 | 0.371  0.262 | 0.041  0.899 | 0.788  0.002 | 0.949  0.000 | 0.935  0.000 | 0.958  0.000 | 0.116  0.720 | *asp* |
|  |  |  |  |  |  |  |  |  |  |  |  | 1 | -0.336  0.312 | -0.305  0.363 | 0.443  0.172 | 0.779  0.005 | 0.764  0.006 | 0.915  0.000 | 0.908  0.000 | 0.648  0.031 | *glcD* |
|  |  |  |  |  |  |  |  |  |  |  |  |  | 1 | 0.978  0.000 | 0.096  0.779 | 0.077  0.821 | -0.350  0.292 | -0.273  0.416 | -0.188  0.580 | -0.74  0.828 | *glnA* |
|  |  |  |  |  |  |  |  |  |  |  |  |  |  | 1 | 0.108  0.752 | 0.151  0.658 | -0.287  0.392 | -0.191  0.575 | -0.133  0.697 | -0.015  0.965 | *hetR* |
|  |  |  |  |  |  |  |  |  |  |  |  |  |  |  | 1 | 0.333  0.291 | -0.084  0.796 | 0.028  0.931 | 0.119  0.712 | 0.666  0.018 | *nifH* |
|  |  |  |  |  |  |  |  |  |  |  |  |  |  |  |  | 1 | 0.592  0.043 | 0.811  0.001 | 0.864  0.000 | 0.542  0.069 | *rbcL* |
|  |  |  |  |  |  |  |  |  |  |  |  |  |  |  |  |  | 1 | 0.885  0.000 | 0.895  0.000 | -0.126  0.695 | *rca* |
|  |  |  |  |  |  |  |  |  |  |  |  |  |  |  |  |  |  | 1 | 0.963  0.000 | 0.119  0.712 | *psaB* |
|  |  |  |  |  |  |  |  |  |  |  |  |  |  |  |  |  |  |  | 1 | 0.258  0.418 | *psaC* |
|  |  |  |  |  |  |  |  |  |  |  |  |  |  |  |  |  |  |  |  | 1 | *psbA* |

S1ii-

| *bicA1* | *bicA2* | *ccmM* | *ccmK2* | *ccmK3* | *ndhF4* | *ndhD4* | *ndhL* | *chpX* | *atpB* | *sod* | *asp* | *glcD* | *glnA* | *hetR* | *nifH* | *rbcL* | *rca* | *psaB* | *psaC* | *psbA* |  |
| --- | --- | --- | --- | --- | --- | --- | --- | --- | --- | --- | --- | --- | --- | --- | --- | --- | --- | --- | --- | --- | --- |
| 1 | 0.957  0.000 | 0.736  0.004 | 0.841  0.000 | 0.875  0.000 | 0.560  0.046 | 0.620  0.031 | 0.551  0.051 | 0.605  0.037 | 0.717  0.006 | 0.716  0.006 | 0.708  0.007 | 0.787  0.001 | 0.284  0.397 | 0.368  0.265 | 0.791  0.001 | 0.768  0.002 | 0.497  0.098 | 0.551  0.051 | 0.758  0.003 | 0.715  0.006 | *bicA1* |
|  | 1 | 0.678  0.015 | 0.840  0.001 | 0.946  0.000 | 0.454  0.138 | 0.501  0.117 | 0.534  0.074 | 0.525  0.097 | 0.628  0.029 | 0.614  0.034 | 0.789  0.002 | 0.684  0.014 | 0.174  0.631 | 0.213  0.555 | 0.760  0.004 | 0.903  0.000 | 0.721  0.008 | 0.691  0.013 | 0.683  0.014 | 0.642  0.024 | *bicA2* |
|  |  | 1 | 0.958  0.00 | 0.802  0.001 | 0.946  0.000 | 0.945  0.000 | 0.811  0.001 | 0.957  0.000 | 0.973  0.000 | 0.954  0.000 | 0.162  0.598 | 0.913  0.00 | 0.723  0.012 | 0.816  0.002 | 0.796  0.001 | 0.795  0.001 | 0.467  0.107 | 0.664  0.013 | 0.980  0.000 | 0.963  0.000 | *ccmM* |
|  |  |  | 1 | 0.911  0.000 | 03.857  0.000 | 0.872  0.000 | 0.785  0.001 | 0.899  0.000 | 0.932  0.000 | 0.910  0.000 | 0.379  0.202 | 0.899  0.000 | 0.591  0.055 | 0.677  0.022 | 0.835  0.000 | 0.879  0.000 | 0.564  0.045 | 0.706  0.007 | 0.956  0.000 | 0.937  0.000 | *ccmK2* |
|  |  |  |  | 1 | 0.608  0.028 | 0.623  0.31 | 0.631  0.021 | 0.684  0.014 | 0.745  0.003 | 0.720  0.006 | 0.607  0.028 | 0.707  0.007 | 0.388  0.238 | 0.412  0.208 | 0.883  0.000 | 0.910  0.000 | 0.589  0.034 | 0.626  0.022 | 0.781  0.002 | 0.777  0.002 | *ccmK3* |
|  |  |  |  |  | 1 | 0.963  0.000 | 0.766  0.002 | 0.967  0.000 | 0.966  0.000 | 0.942  0.000 | -0.105  0.734 | 0.882  0.000 | 0.797  0.003 | 0.904  0.000 | 0.663  0.013 | 0.633  0.020 | 0.333  0.267 | 0.555  0.049 | 0.944  0.000 | 0.944  0.000 | *ndhF4* |
|  |  |  |  |  |  | 1 | 0.837  0.001 | 0.983  0.000 | 0.942  0.000 | 0.975  0.000 | -0.021  0.947 | 0.953  0.000 | 0.795  0.003 | 0.898  0.000 | 0.697  0.012 | 0.700  0.011 | 0.435  0.157 | 0.693  0.012 | 0.963  0.000 | 0.935  0.000 | *ndhD4* |
|  |  |  |  |  |  |  | 1 | 0.955  0.000 | 0.790  0.001 | 0.892  0.000 | 0.143  0.624 | 0.838  0.000 | 0.650  0.031 | 0.717  0.013 | 0.762  0.002 | .0705  0.007 | 0.310  0.303 | 0.612  0.026 | 0.855  0.000 | 0.820  0.001 | *ndhL* |
|  |  |  |  |  |  |  |  | 1 | 0.949  0.000 | 0.975  0.000 | -0.034  0.915 | 0.921  0.000 | 0.837  0.001 | 0.912  0.000 | 0.761  0.004 | 0.753  0.005 | 0.479  0.115 | 0.708  0.010 | 0.965  0.000 | 0.955  0.000 | *chpX* |
|  |  |  |  |  |  |  |  |  | 1 | 0.965  0.000 | 0.114  0.710 | 0.926  0.000 | 0.694  0.018 | 0.814  0.002 | 0.756  0.003 | 0.719  0.006 | 0.385  0.194 | 0.575  0.040 | 0.983  0.000 | 0.988  0.000 | *atpB* |
|  |  |  |  |  |  |  |  |  |  | 1 | 0.125  0.683 | 0.967  0.000 | 0.768  0.006 | 0.865  0.001 | 0.812  0.001 | 0.754  0.003 | 0.44  0.171 | 0.651  0.016 | 0.983  0.000 | 0.969  0.000 | *sod* |
|  |  |  |  |  |  |  |  |  |  |  | 1 | 0.272  0.369 | -0.422  0.196 | -0.387  0.240 | 0.382  0.198 | 0.524  0.066 | 0.412  0.162 | 0.333  0.267 | 0.205  0.501 | 0.149  0.627 | *asp* |
|  |  |  |  |  |  |  |  |  |  |  |  | 1 | 0.642  0.033 | 0.764  0.006 | 0.743  0.004 | 0.760  0.003 | 0.464  0.110 | 0.703  0.0074 | 0.964  0.000 | 0.927  0.000 | *glcD* |
|  |  |  |  |  |  |  |  |  |  |  |  |  | 1 | 0.969  0.000 | 0.679  0.022 | 0.484  0.132 | 0.297  0.375 | 0.507  0.112 | 0.684  0.020 | 0.697  0.017 | *glnA* |
|  |  |  |  |  |  |  |  |  |  |  |  |  |  | 1 | 0.658  0.028 | 0.485  0.130 | 0.255  0.448 | 0.505  0.113 | 0.798  0.003 | 0.799  0.003 | *hetR* |
|  |  |  |  |  |  |  |  |  |  |  |  |  |  |  | 1 | 0.846  0.000 | 0.470  0.105 | 0.588  0.035 | 0.0793  0.001 | 0.802  0.001 | *nifH* |
|  |  |  |  |  |  |  |  |  |  |  |  |  |  |  |  | 1 | 0.7878  0.001 | 0.862  0.000 | 0.797  0.00011 | 0.783  0.002 | *rbcL* |
|  |  |  |  |  |  |  |  |  |  |  |  |  |  |  |  |  | 1 | 0.912  0.000 | 0.462  0.112 | 0.464  0.110 | *rca* |
|  |  |  |  |  |  |  |  |  |  |  |  |  |  |  |  |  |  | 1 | 0.676  0.11 | 0.642  0.018 | *psaB* |
|  |  |  |  |  |  |  |  |  |  |  |  |  |  |  |  |  |  |  | 1 | 0.984  0.000 | *psaC* |
|  |  |  |  |  |  |  |  |  |  |  |  |  |  |  |  |  |  |  |  | 1 | *psbA* |

S1iii-

| *bicA1* | *bicA2* | *ccmM* | *ccmK2* | *ccmK3* | *ndhF4* | *ndhD4* | *ndhL* | *chpX* | *atpB* | *sod* | *asp* | *glcD* | *glnA* | *hetR* | *nifH* | *rbcL* | *rca* | *psaB* | *psaC* | *psbA* |  |
| --- | --- | --- | --- | --- | --- | --- | --- | --- | --- | --- | --- | --- | --- | --- | --- | --- | --- | --- | --- | --- | --- |
| 1 | 0.449  0.143 | 0.522  0.082 | 0.506  0.093 | 0.356  0.257 | 0.363  0.246 | 0.353  0.260 | 0.223  0.487 | 0.428  0.165 | 0.107  0.740 | 0.678  0.015 | -0.196  0.541 | 0.340  0.280 | 0.243  0.472 | 0.104  0.749 | 0.093  0.773 | 0.330  0.298 | 0.028  0.930 | 0.358  0.253 | 0.732  0.007 | 0.297  0.349 | *bicA1* |
|  | 1 | 0.387  0.214 | 0.342  0.277 | 0.484  0.111 | 0.449  0.143 | 0.612  0.035 | 0.486  0.109 | 0.139  0.666 | 0.212  0.509 | 0.460  0.133 | 0.359  0.252 | 0.219  0.494 | -0.135  0.693 | -0.218  0.495 | 0.295  0.351 | 0.360  0.251 | 0.002  0.995 | -0.058  0.859 | 0.483  0.111 | 0.208  0.516 | *bicA2* |
|  |  | 1 | 0.949  0.000 | 0.781  0.003 | 0.872  0.00 | 0.842  0.001 | 0.149  0.644 | 0.892  0.000 | 0.595  0.041 | 0.486  0.110 | -0.144  0.655 | 0.437  0.156 | 0.695  0.017 | 0.688  0.013 | 0.685  0.014 | 0.831  0.001 | -0.206  0.520 | -0.202  0.528 | 0.577  0.050 | 0.771  0.003 | *ccmM* |
|  |  |  | 1 | 0.816  0.001 | 0.910  0.000 | 0.875  0.000 | 0.252  0.430 | 0.957  0.000 | 0.702  0.011 | 0.639  0.025 | 0.045  0.890 | 0.582  0.047 | 0.691  0.018 | 0.704  0.011 | 0.833  0.001 | 0.936  0.000 | -0.043  0.894 | -0.289  0.362 | 0.583  0.046 | 0.921  0.000 | *ccmK2* |
|  |  |  |  | 1 | 0.948  0.00 | 0.902  0.000 | 0.683  0.014 | 0.756  0.004 | 0.881  0.000 | 0.648  0.023 | 0.399  0.199 | 0.354  0.259 | 0.252  0.455 | 0.283  0.372 | 0.812  0.001 | 0.748  0.005 | -0.115  0.123 | -0..378  0.225 | 0.614  0.034 | 0.705  0.010 | *ccmK3* |
|  |  |  |  |  | 1 | 0.923  0.000 | 0.481  0.113 | 0.847  0.001 | 0..89  0.000 | 0.598  0.040 | 0.236  0.460 | 0.384  0.218 | 0.477  0.136 | 0.487  0.108 | 0.872  0.000 | 0.839  0.001 | -0.202  0.530 | -0.434  0.159 | 0.590  0.044 | 0.827  0.001 | *ndhF4* |
|  |  |  |  |  |  | 1 | 0.472  0.121 | 0.755  0.005 | 0.725  0.008 | 0.583  0.047 | 0.362  0.247 | 0.473  0.120 | 0.427  0.190 | 0.418  0.177 | 0.870  0.000 | 0.885  0.000 | -0.055  0.866 | -0.507  0.093 | 0.467  0.126 | 0.798  0.002 | *ndhD4* |
|  |  |  |  |  |  |  | 1 | 0.239  0.455 | 0.592  0.043 | 0.609  0.036 | 0.765  0.004 | 0.075  0.817 | -0.479  0.136 | -0.410  0.186 | 0.411  0.184 | 0.251  0.431 | 0.086  0.791 | -0.150  0.641 | 0.509  0.091 | 0.212  0.508 | *ndhL* |
|  |  |  |  |  |  |  |  | 1 | 0.686  0.014 | 0.571  0.053 | -0.010  0.976 | 0.476  0.118 | 0.681  0.021 | 0.731  0.007 | 0.775  0.003 | 0.868  0.000 | -0.103  0.750 | -0.249  0.434 | 0.567  0.054 | 0.886  0.000 | *chpX* |
|  |  |  |  |  |  |  |  |  | 1 | 0.547  0.066 | 0.406  0.190 | 0.299  0.345 | 0.280  0.404 | 0.331  0.294 | 0.843  0.001 | 0.666  0.018 | -0.101  0.754 | -0.454  0.138 | 0.479  0.115 | 0.726  0.007 | *atpB* |
|  |  |  |  |  |  |  |  |  |  | 1 | 0.400  0.198 | 0.702  0.011 | 0.178  0.600 | 0.169  0.599 | 0.564  0.056 | 0.617  0.033 | 0.342  0.277 | 0.098  0.763 | 0.725  0.008 | 0.635  0.026 | *sod* |
|  |  |  |  |  |  |  |  |  |  |  | 1 | 0.239  0.454 | -0.462  0.152 | -0.381  0.222 | 0.478  0.116 | 0.247  0.439 | 0.374  0.232 | -0.408  0.191 | -0.038  0.906 | 0.222  0.906 | *asp* |
|  |  |  |  |  |  |  |  |  |  |  |  | 1 | 0.530  0.093 | 0.525  0.079 | 0.587  0.045 | 0.707  0.010 | 0.672  0.017 | 0.077  0.812 | 0.241  0.451 | 0.704  0.011 | *glcD* |
|  |  |  |  |  |  |  |  |  |  |  |  |  | 1 | 0.990  0.000 | 0.518  0.103 | 0.679  0.022 | -0.059  0.863 | -0.150  0.660 | 0.100  0.771 | 0.703  0.016 | *glnA* |
|  |  |  |  |  |  |  |  |  |  |  |  |  |  | 1 | 0.543  0.088 | 0.690  0.013 | -0.023  0.944 | -0.160  0.619 | 0.094  0.771 | 0.730  0.007 | *hetR* |
|  |  |  |  |  |  |  |  |  |  |  |  |  |  |  | 1 | 0.915  0.000 | 0.114  0.724 | -0.594  0.042 | 0.278  0.3881 | 0.929  0.000 | *nifH* |
|  |  |  |  |  |  |  |  |  |  |  |  |  |  |  |  | 1 | 0.195  0.544 | -0.371  0.236 | 0.394  0.205 | 0.960  0.000 | *rbcL* |
|  |  |  |  |  |  |  |  |  |  |  |  |  |  |  |  |  | 1 | 0.311  0.324 | -0.085  0.739 | 0.152  0.637 | *rca* |
|  |  |  |  |  |  |  |  |  |  |  |  |  |  |  |  |  |  | 1 | 0.261  0.413 | -0.403  0.194 | *psaB* |
|  |  |  |  |  |  |  |  |  |  |  |  |  |  |  |  |  |  |  | 1 | 0.386  0.216 | *psaC* |
|  |  |  |  |  |  |  |  |  |  |  |  |  |  |  |  |  |  |  |  | 1 | *psbA* |

S1iv-

| *bicA1* | *bicA2* | *ccmM* | *ccmK2* | *ccmK3* | *ndhF4* | *ndhD4* | *ndhL* | *chpX* | *atpB* | *sod* | *asp* | *glcD* | *glnA* | *hetR* | *nifH* | *rbcL* | *rca* | *psaB* | *psaC* | *psbA* |  |
| --- | --- | --- | --- | --- | --- | --- | --- | --- | --- | --- | --- | --- | --- | --- | --- | --- | --- | --- | --- | --- | --- |
| 1 | 0.937  0.000 | 0.923  0.00 | 0.953  0.00 | 0.681  0.015 | 0.416  0.179 | 0.178  0.580 | 0.862  0.000 | 0.088  0.787 | 0.462  0.130 | 0.446  0.146 | 0.312  0.324 | 0.346  0.271 | 0.025  0.941 | 0.007  0.984 | 0.230  0.472 | 0.317  0.316 | 0.058  0.857 | 0.289  0.363 | 0.892  0.000 | 0.327  0.300 | *bicA1* |
|  | 1 | 0.857  0.000 | 0.858  0.000 | 0.574  0.051 | 0.524  0.080 | 0.235  0.463 | 0.738  0.002 | 0.028  0.931 | 0.372  0.234 | 0.412  0.184 | 0.290  0.360 | 0.381  0.221 | 0.122  0.722 | 0.058  0.866 | 0.239  0.454 | 0.328  0.298 | 0.016  0.960 | 0.114  0.723 | 0.748  0.005 | 0.324  0.304 | *bicA2* |
|  |  | 1 | 0.979  0.000 | 0.767  0.004 | 0.497  0.101 | 0.318  0.313 | 0.638  0.026 | 0.355  0.257 | 0.369  0.238 | 0.323  0.306 | 0.113  0.728 | 0.253  0.0428 | 0.334  0.316 | 0.360  0.276 | 0.334  0.289 | 0.420  0.174 | -0.004  0.991 | 0.281  0.377 | 0.806  0.002 | 0.105  0.745 | *ccmM* |
|  |  |  | 1 | 0.792  0.002 | 0.427  0.166 | 0.256  0.421 | 0.736  0.006 | 0.333  0.290 | 0.402  0.195 | 0.396  0.202 | 0.176  0.585 | 0.285  0.369 | 0.169  0.620 | 0.224  0.507 | 0.339  0.282 | 0.384  0.218 | 0.083  0.796 | 0.370  0.237 | 0.870  0.000 | 0.192  0.550 | *ccmK2* |
|  |  |  |  | 1 | 0.518  0.084 | 0.561  0.058 | 0.582  0.047 | 0.637  0.026 | 0.579  0.048 | 0.700  0.011 | 0.433  0.160 | 0.567  0.055 | 0.177  0.603 | 0.367  0.267 | 0.671  0.017 | 0.726  0.007 | 0.406  0.191 | 0.670  0.017 | 0.706  0.010 | 0.420  0.175 | *ccmK3* |
|  |  |  |  |  | 1 | 0.913  0.000 | 0.264  0.407 | 0.507  0.092 | 0.378  0.225 | 0.549  0.065 | 0.462  0.130 | 0.582  0.047 | 0.404  0.217 | 0.491  0.15 | 0.595  0.041 | 0.799  0.002 | 0.439  0.153 | 0.219  0.493 | 0.265  0.405 | 0.465  0.128 | *ndhF4* |
|  |  |  |  |  |  | 1 | 0.068  0.833 | 0.675  0.016 | 0.426  0.167 | 0.586  0.045 | 0.478  0.116 | 0.604  0.037 | 0.409  0.212 | 0.575  0.064 | 0.757  0.004 | 0.898  0.000 | 0.567  0.054 | 0.37  0.231 | 0.132  0.682 | 0.476  0.118 | *ndhD4* |
|  |  |  |  |  |  |  | 1 | -0.092  0.775 | 0.605  0.037 | 0.644  0.024 | 0.619  0.032 | 0.482  0.113 | -0.437  0.179 | -0.421  0.197 | 0.234  0.464 | 0.233  0.466 | 0.211  0.510 | 0.376  0.228 | 0.885  0.000 | 0.655  0.21 | *ndhL* |
|  |  |  |  |  |  |  |  | 1 | 0.022  0.946 | 0.319  0.313 | -0.005  0.988 | 0.266  0.404 | 0.358  0.279 | 0.701  0.016 | 0.616  0.033 | 0.621  0.031 | 0.477  0.117 | 0.438  0.155 | 0.107  0.741 | 0.054  0.867 | *chpX* |
|  |  |  |  |  |  |  |  |  | 1 | 0.661  0.019 | 0.794  0.002 | 0.465  0.127 | -0.160  0.638 | -0.183  0.589 | 0.648  0.023 | 0.524  0.080 | 0.2328  0.456 | 0.509  0.091 | 0.685  0.014 | 0.691  0.013 | *atpB* |
|  |  |  |  |  |  |  |  |  |  | 1 | 0.886  0.000 | 0.912  0.000 | -0.260  0.440 | -0.109  0.749 | 0.549  0.064 | 0.778  0.003 | 0.779  0.003 | 0.766  0.004 | 0.499  0.99 | 0.896  0.000 | *sod* |
|  |  |  |  |  |  |  |  |  |  |  | 1 | 0.788  0.002 | -0.434  0.182 | -0.389  0.237 | 0.457  0.135 | 0.593  0.042 | 0.578  0.049 | 0.561  0.058 | 0.449  0.144 | 0.959  0.000 | *asp* |
|  |  |  |  |  |  |  |  |  |  |  |  | 1 | -0.100  0.769 | -0.008  0.982 | 0.388  0.212 | 0.805  0.002 | 0.733  0.007 | 0.652  0.022 | 0.289  0.362 | 0.800  0.002 | *glcD* |
|  |  |  |  |  |  |  |  |  |  |  |  |  | 1 | 0.912  0.000 | 0.151  0.657 | 0.368  0.266 | -0.219  0.518 | -0.136  0.691 | -0.205  0.545 | -0.532  0.092 | *glnA* |
|  |  |  |  |  |  |  |  |  |  |  |  |  |  | 1 | 0.344  0.3000 | 0.503  0.114 | 0.013  0.970 | 0.029  0.932 | -0.174  0.809 | -0.430  0.187 | *hetR* |
|  |  |  |  |  |  |  |  |  |  |  |  |  |  |  | 1 | 0.685  0.014 | 0.413  0.182 | 0.415  0.179 | 0.384  0.218 | 0.488  0.108 | *nifH* |
|  |  |  |  |  |  |  |  |  |  |  |  |  |  |  |  | 1 | 0.677  0.016 | 0.657  0.020 | 0.275  0.387 | 0.560  0.059 | *rbcL* |
|  |  |  |  |  |  |  |  |  |  |  |  |  |  |  |  |  | 1 | 0.762  0.004 | 0.079  0.808 | 0.661  0.019 | *rca* |
|  |  |  |  |  |  |  |  |  |  |  |  |  |  |  |  |  |  | 1 | 0.438  0.154 | 0.525  0.080 | *psaB* |
|  |  |  |  |  |  |  |  |  |  |  |  |  |  |  |  |  |  |  | 1 | 0.441  0.151 | *psaC* |
|  |  |  |  |  |  |  |  |  |  |  |  |  |  |  |  |  |  |  |  | 1 | *psbA* |
